# Serum Protein Profiling of Patients at Risk to Develop Gastric Disease Based on a DSC Test

**DOI:** 10.3390/ijms27104464

**Published:** 2026-05-16

**Authors:** Ombretta Repetto, Filippo Sperti, Mariangela De Zorzi, Veronica Paduano, Stefano Realdon, Agostino Steffan, Renato Cannizzaro, Valli De Re

**Affiliations:** 1 Experimental and Clinical Pharmacology, Centro di Riferimento Oncologico di Aviano (CRO) IRCCS, 33081 Aviano, Italy; 2 Immunopathology and Cancer Biomarkers, Centro di Riferimento Oncologico di Aviano (CRO) IRCCS, 33081 Aviano, Italy; 3 Oncological Gastroenterology, Centro di Riferimento Oncologico di Aviano (CRO) IRCCS, 33081 Aviano, Italy; 4 Department of Medical, Surgical and Health Sciences, University of Trieste, 34127 Trieste, Italy

**Keywords:** biomarker, gastric cancer, gastritis, mass spectrometry, proteomics, screening, beta-2-microglobulin

## Abstract

At present, the gold standard for gastric cancer (GC) confirmation relies mostly on histopathology, an invasive procedure. Noninvasive detection methods using serum for large-scale screening may be useful for the early diagnosis of GC. *Helicobacter pylori* (HP) infection and chronic atrophic gastritis are major GC risk factors. We recently developed a noninvasive test called the DSC test-based on the patient’s age, sex, their serum PGI and PGII, anti-HP immunoglobulin (IgG), and gastrin G17 levels-predicting GC risk as low (score 0, S0) or high (score 2, S2). The comparative investigation at the serum protein level of the two different patient groups detected by our DCS test (S0 and S2) may undoubtedly help to identify gastric disease-dependent proteins, resulting from bacterial infection or gastric mucosa inflammation, as well as get better insight into the molecular scenario associated with pre-cancerous conditions. We used an untargeted liquid chromatography–tandem mass spectrometry (LC-MS/MS)-based proteomic profiling approach, followed by univariate statistical analysis to compare the different DSC groups across two patient cohorts (exploratory and validation). Significantly differentially abundant proteins differing more than 1.5-fold between S0 and S2 groups were selected and validated, and their putative role(s) in gastritis and GC were discussed. In both the exploratory and the validation cohorts, four proteins (beta-2-microglobulin, EGF-containing fibulin-like extracellular matrix protein 1, complement factor D, and cystatin-C) were more abundant, while two (sex hormone-binding globulin and pregnancy zone protein) were less abundant in the sera of S2 individuals (|fold change| ≥ 0.6, *p* < 0.05, *t*-test). The higher presence of beta-2-microglobulin (B2M) and the lower content of pregnancy zone protein (PZP) in S2 sera were validated by immunoblotting. Replacing age and sex in our DSC model with two specific candidate biomarkers can lead to a refined, albeit modest, improvement in classification accuracy. This study identified a proteomic signature that was differentially associated with the sera of patients with a different risk to develop advanced atrophy/GC according to the DSC test. Moving from a demographic model to a proteomic-driven model can better reflect the personalized biology of pathological processes associated with DSC.

## 1. Introduction

Gastric cancer (GC) is a highly aggressive and complex malignancy representing a global health problem: it is the fifth most commonly diagnosed cancer and the fifth leading cause of cancer-related death worldwide in 2022 [1]. GC is classified into two main histological subtypes, with distinct characteristics: diffuse (poorly cohesive cells, including signet ring cells and linitis plastic) and intestinal (glandular growth pattern). Intestinal-type histology represents the large majority of GC worldwide [2,3]. Diffuse GC is largely driven by mutations in pathways regulating cell–extracellular matrix interactions, while intestinal GC typically arises from chronic atrophic gastritis, primarily triggered by *Helicobacter pylori* (HP), a class I carcinogen, through a succession of a well-delineated sequential series of precancerous steps, known as the “Correa cascade”, the gastric advanced atrophy predisposing to intestinal metaplasia, dysplasia and, finally, adenocarcinoma [4,5]. While HP and premalignant lesions are known risk factors, most affected individuals do not develop gastric cancer. Therefore, clear guidelines on who to screen, when to start, and which methods to use are still lacking.

The etiology of GC is notably multifactorial, with its development arising from an interplay of several risk factors: several modifiable (HP, obesity, diet, smoking and alcohol consumption) and nonmodifiable (gene susceptibility, age, family history and male gender) factors may interact and impact GC risk/progression, thus suggesting potential preventive strategies [6].

At present, primary (HP test-and-treat) and secondary (upper gastrointestinal endoscopy and histology) preventive strategies for GC are available. In particular, the gold standard for screening asymptomatic individuals or surveillance of high-risk subgroups for GC is upper endoscopy with biopsy, despite its invasive nature, which detects early-stage cancer or premalignant gastric lesions (atrophy, dysplasia or intestinal metaplasia) [7]. In this context, the endoscopic management of gastric precancerous lesions has been recently updated in the MAPS II guidelines [8].

In Western countries, GC is frequently diagnosed at an advanced stage as incurable/metastatic disease with a poor prognosis, so that its early diagnosis becomes crucial in the improvement of therapy efficacy and long-term patient survival [9]. In recent years, evidence has strongly suggested that despite the availability of effective diagnostic tools for GC, there is no ‘one-size-fits-all’ screening method. Strategies must therefore be adapted to regional contexts, tailoring approaches for high-risk populations versus those in lower-risk areas [10,11,12,13]. At present, national population-wide screening programs for GC have been implemented in only few East Asian countries, at a high prevalence of GC [14]. In Western Europe, at a low prevalence of GC, routine endoscopic screening for GC is currently not suitable for large-scale screening protocols, due to the low incidence of GC and the high cost of the procedure [15]. Most Western nations, including Italy, recommend a personalized or risk-stratified screening strategy, in which endoscopy is reserved exclusively for high-risk subjects [13]. In this context, current efforts to identify high-risk populations include several rigorous testing protocols. The clinical utility of serum-based markers like CEA, CA 19-9, and CA 72-4 remains limited in screening and early diagnosis in GC, as they do not meet the required thresholds for sensitivity and specificity [14]. However, serology may have an important role in GC prevention, in particular with the so-called GastroPanel^®^ test and its simultaneous detection of pepsinogen type I and II (PGI and PGII), PGI/PGII ratio, gastrin-17 (G17) and anti-HP IgG antibodies. This test has been originally conceived to diagnose diffuse atrophic gastritis [16]. However, since the 1990s, these markers have also been utilized to identify patients who are at high risk for GC (characterized by reduced PGI and PGI/PGII values). Although their sensitivity remains low, they help to identify candidates who may require an upper gastrointestinal endoscopy [17,18,19,20].

Recently, we have developed a model—called the DSC test—based on the patient’s age, sex, their serum PGI and PGII, anti-HP immunoglobulin (IgG), and G17 levels, to individuate patients who are at higher risk of developing GC in a medium-risk GC Italian area early [21]. The DSC model grouped patients based on risk classes to develop advanced gastric atrophy or GC into negative (score 0; no risk), neutral (score 1; low-risk), and positive (score 2; high risk) results. In this work, we employed liquid chromatography–tandem mass spectrometry (LC-MS/MS) to identify the serum proteome signature in individuals with a positive DSC test (score 2), which may help us to get better insight into the molecular phenotypes of our DSC-selected subjects who are at risk for gastric pathology and, indirectly, improve the diagnostic utility of our test.

## 2. Results

### 2.1. Demographic Characteristics and Hematological Parameters

Table 1 shows the characteristics of patients in the discovery (*n* = 20) and validation (*n* = 80) cohorts. None of the laboratory-tested parameters (HP IgG, PGI, PGII, PGI/PGII, G17) differed significantly between the two cohorts. The cohorts were also comparable in gender distribution (~30% male). However, a statistically significant difference was observed in age distribution: individuals aged 50–70 years were more prevalent in the discovery cohort (40%), whereas subjects aged <50 years were more frequent in the validation cohort (51%) than in the discovery one (25%) (*p*-test = 0.045). Individuals classified as S0 were significantly younger than those in the S2 group. In a smaller subset of 23 patients with available histological evaluation, 17 showed no significant lesions, 4 presented chronic atrophy and/or intestinal metaplasia and 2 cases were classified as “advanced lesions” (one with severe atrophy, Olga stage ≥ 3, and one GC).

### 2.2. Plasma Protein Profiling

**Figure 1:**
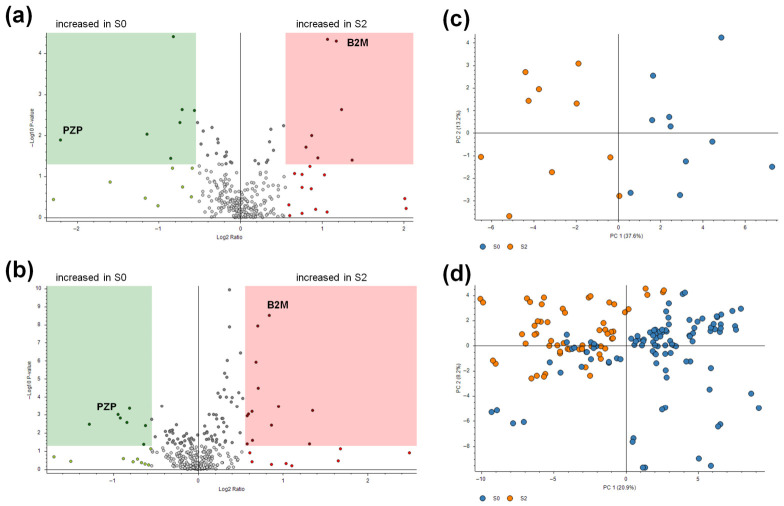
Volcano plot showing proteomic data by DSC score status (S2 vs. S0) in the discovery and exploratory cohorts (**a**,**b**). Principal component analysis (PCA) of the proteins identified by LC-MS/MS analysis (normalized protein abundances) as being differently expressed by the DSC score groups in the discovery and exploratory cohorts (**c**,**d**). In both Volcano plots (**a**,**b**), Log2 transformed abundance ratios for each protein are plotted on the *x*-axis. Negative log10 transformed *p*-values are plotted on the *y*-axis. Proteins significantly more abundant in S2 patient sera (red circles) are evidenced by the red area (abundance ratio S2/S0 (log2) ≥ 0.6 and *p*-value ≤ 0.05), while those more abundant in S0 patient sera (green circles) are evidenced by the green area (abundance ratio S2/S0 (log2) ≤ −0.6 and *p*-value ≤ 0.05). The proteins with differences that were not statistically significant between S2 and S0 are shown in gray. Positions of B2M and PZPs are evidenced on the graphs. In both PCA (**c**,**d**), each circle represents an individual patient’s protein profile belonging to the S0 (blue circles) or S2 (orange circles) DSC score status. In (**d**), both technical replicates for each patient are shown. B2M, beta-2-microglobulin and PZP, pregnancy zone protein.

In Part I of the study (discovery phase), serum samples from patients S0 and S2 were analyzed using label-free LC-MS/MS. Based on (|FC(log2)| ≥ 0.6, *t*-test *p*-value < 0.05), a total of 14 differentially abundant proteins were identified (Table 2; Supplementary Table S1; Figure 1a). In particular, quantitative comparisons between the S2 and S0 groups identified seven proteins that were significantly more abundant in the serum of patients at high risk of gastric disease, and seven proteins that were more abundant in the serum of individuals at low risk (*t*-test, *p*-value < 0.05).

In Part II of the study (validation phase), label-free LC-MS/MS analysis identified a total of 21 proteins differing in abundance—based on FC and *p*-values—between the S2 and S0 groups in our validation cohort (Table 3; Supplementary Table S2; Figure 1b).

Principal component analysis (PCA) applied to serum protein profiling was effective at separating patient groups based on risk levels S0 and S2, albeit with marked biological variability among patients in both the discovery and validation (Figure 1c,d).

The following six proteins were confirmed as being differentially abundant in S2 versus S0 groups in both discovery and validation cohorts: complement factor D (CFD), beta-2-microglobulin (B2M), EGF-containing fibulin-like extracellular matrix protein 1 (EFEMP1), and cystatin-C (CST3), which were more abundant in S2 groups; and sex hormone-binding globulin (SHBG) and pregnancy zone protein (PZP), which were less abundant in S2 individuals.

**Figure 2:**
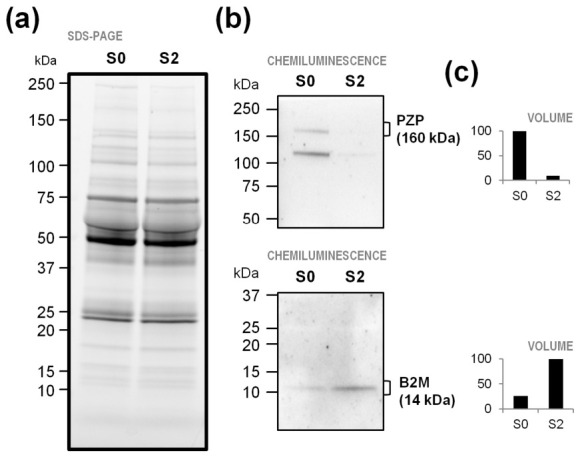
Immunochemical detection of two differentially abundant serum proteins, indicated by gene names on the right. Samples are pools of serum proteins from individuals with DSC scores 0 and 2 of the validation cohort. (**a**) Stain-free gel acquired before transfer as control for equal loading of protein extracts. (**b**) Cross-reactive bands against PZP or B2M antibodies. (**c**) Band volume intensities from immunoblotting. B2M, beta-2-microglobulin and PZP, pregnancy zone protein.

Differences in the abundance of B2M and PZP were examined by immunoblotting serum proteins from the S0 and S2 groups of the validation cohort (pools of 51 and 29 samples, respectively). The higher level of B2M as well as the lower level of PZP was confirmed in S2 serum compared to S0 (Figure 2).

A significant correlation was observed between the serum relative abundance of B2M and age (r = 0.659, *p* = 0.002), and between the PZP levels and sex (r = −0.205, *p* < 0.001), respectively (Table 4).

At the tissue level, PCA of gastric protein profiles discriminated between S2 and S0 patients, suggesting that our DSC classification reflects underlying differences in gastric tissue protein expression patients from (Supplementary Figure S1). There was a significant increase in PZP abundance in the gastric tissues of S2 patients (FC S2/S0 (log2) = 2.50; *p*-value = 0.013), whereas no significant difference was observed in B2M abundance levels between the S2 and S0 groups (FC S2/S0 (log2) = −0.13, *p*-value = 0.30).

In this cohort of patients older than 55 years, B2M abundance in gastric tissue did not significantly correlate with age (Supplementary Figure S2a), while PZP abundance was significantly associated with sex in S2 subjects (*p* < 0.001, ANOVA test; Supplementary Figure S2b). Moreover, differentially abundant gastric tissue proteins showed distinct inflammatory profiles between the S2 and S0 groups, suggesting that our serological classification is associated with underlying differences in mucosal immune activity (Supplementary Figure S3). In particular, markers of innate immune activation (e.g., defensin alpha 3, DEFA3, and SLAM family member 9, SLAMF9) were relatively elevated in the S0 group, whereas S2 samples exhibited a distinct profile, potentially reflecting more advanced mucosal remodeling, rather than acute inflammation.

### 2.3. Comparison of DSC Classification Accuracy

To assess the clinical utility of B2M and PZP, we compared a model incorporating these candidate biomarkers with a baseline model comprising age and sex; the established serological markers (PGI, PGII, G17, and *H. pylori* IgG) were retained in both models. Both approaches achieved 100% sensitivity and negative predictive values, indicating a strong ability to exclude individuals who were at high risk for GC at gastroscopy (score 2) (Supplementary Table S3). However, the specificity and positive predictive value remained limited, likely reflecting both the exploratory nature of the analysis and the underlying disease prevalence. Overall, the addition of B2M and PZP resulted in a marginal improvement in performance, with slightly higher discrimination (AUC 0.74 vs. 0.71) and overall accuracy compared to the standard DSC model.

Given the potential influence of age on biomarker performance, we further investigate its contribution in a regression model including B2M and PZP instead of age and sex. In this regression model, age remained significantly associated with the outcome (coefficient: 0.6784; Supplementary Figure S4), suggesting that the predictive contribution of B2M and PZP does not fully account for age-related effects.

We then performed a separate ROC-AUC analysis focused on gastric atrophy and mild preneoplastic lesions. In this setting, the model including B2M and PZP showed improved performance discrimination, yielding an AUC of 0.941 compared with 0.853 for the standard model (Supplementary Figure S5). These findings suggest that B2M and PZP may provide additive value for refining risk stratification, particularly for early-stage gastric mucosal alterations. Nevertheless, performance for advanced preneoplastic and neoplastic lesions remained limited (~50%) in both models (Supplementary Table S3).

## 3. Discussion

We recently proposed a noninvasive screening method called the DSC test—based on the patient’s age, sex, serum PGI and PGII, anti-HP IgG, and G17 levels—predicting the risk of developing gastric disease and categorizing patients into low- (score 0, S0) and high-risk (score 2, S2) groups for GC in Italian geographical areas with medium GC risk (Veneto and Friuli-Venezia Giulia) [21]. This test appeared to be valuable as a potential screening strategy that was capable of identifying asymptomatic individuals who require upper gastrointestinal diagnostic endoscopy. Hereby, extending our previous research, we present a study adopting a mass spectrometry-based proteomic approach to identify new candidate markers associated with early GC risk, intended for incorporation into the DSC test to improve its accuracy.

To date, numerous studies have investigated predictive biomarkers for the early detection of stomach cancer or precancerous gastric protein levels [22,23,24]. The identification of circulating biomarkers that were suitable for population screening and early detection of GC yielded significant advancements both in plasma (5-protein panel, [25]; 13-protein panel, [26]; HLA-G, [27]; miscellaneous aspects, such as in [28]; sex hormone-binding globulin [29]) and in serum (ITIH4 [30]; survivin, [31]; and cytokines, [32]). However, these markers—as well as conventional serum markers like CEA and CA19-9 [33]—demonstrate suboptimal sensitivity and positive predictive value. Their limited diagnostic power highlights the need for more sensitive and specific alternatives to reduce GC incidence and mortality.

In differently large clinical cohorts of patients, many studies have reported lists of candidate disease-associated proteins, using different technological platforms, among which mass spectrometry-based proteomics has become a cornerstone of biomedical research, enabling the routine, comprehensive, and high-throughput identification/quantification of proteins in complex biological samples. In particular, the “bottom-up” approach is the dominant method for discovery-driven profiling, bypassing the need for extensive prefractionation by digesting entire proteomes into peptides for liquid chromatography–tandem mass spectrometry (LC-MS/MS) analysis [34].

Our LC-MS/MS-based label-free quantitative proteomic approach succeeded in identifying quantifiable differences in serum protein levels across S0 and S2 patients, in two independent cohorts (discovery and validation). In sera of S2 patients of both cohorts, five proteins were found to be significantly more abundant (complement factor D, CFD; beta-2-microglobulin, B2M; EGF-containing fibulin-like extracellular matrix protein 1, EFEMP1, and cystatin-C, CST3), while two decreased in content (sex hormone-binding globulin, SHBG, and pregnancy zone protein, PZP). Among them, we focused on B2M and PZP, whose differential content was validated by immunoblotting. In particular, B2M and PZP were found to significantly increase and decrease in relative abundance in serum of S2 patients compared with S0.

B2M, a low-molecular-weight house-keeping protein (around 12 kDa), is synthesized by nearly all nucleated cells, including lymphocytes. It is non-covalently linked to the α-chain of major histocompatibility complex class I (MHC-I) molecules, the B2M subunit facilitating antigenic peptide presentation to natural killer cells and CD8+ cytotoxic T lymphocytes but also reinforcing MHC-I structural integrity [35,36]. Mainly metabolized in the glomerulus after its dissociation from the MHC-I molecule and excreted by the kidneys, this immune surveillance molecule is a sensitive indicator of proximal tubular dysfunction [37]. In certain diseases, B2M is either cleared less efficiently or produced more rapidly, leading to elevated levels in the blood; at the same time, because it lacks covalent bonds to MHC-I and direct anchoring to cell membrane, B2M can detach as a free soluble molecule circulating in the extracellular fluid [38]. Soluble B2M is present in bodily fluids, such as serum, with stable concentrations and at low levels under normal physiological conditions [25], while elevated serum B2M levels have been associated with a variety of malignancies, including certain cancers of the blood and bone marrow (i.e., myeloma [39], Hodgkin disease [40], Burkitt lymphoma [41], chronic lymphocytic leukemia [42], and diffuse large B-cell lymphoma, DLBCL [43]) and non-hematological cancers (i.e., oral squamous cell carcinoma [44]; prostate [45]; small cell lung [46]; glioma [47]), in most of which circulating B2M appeared as a useful biomarker for clinical diagnosis, follow-up and prognosis.

Our observed higher B2M levels in S2 patients may come from a higher inflammatory status, growing evidence emerging about the relationship between circulating B2M and systemic inflammation, high cell turnover and immune activation. Interestingly, we found an association between serum B2M abundance and age, in accordance with previous studies reporting increased circulating B2M levels with aging [48,49]. In this context, the higher B2M levels observed in S2 patients at increased risk of severe gastric disease are also consistent with the DSC screening test, as GC incidence in particular is strongly associated with aging [50]. In contrast, no correlation was observed between the serum B2M level and the serological parameters (PGI, PGII, PGR, G17, and HP IgG). Similarly, in Yeniova et al. (2014)’s work investigating 120 patients (70.8% HP positive of which 27.5% CagA positive), the serum B2M level did not correlate with HP infection severity and intensity according to the Sydney classification and CagA status [51]. Comparable serum B2M levels in the HP and control groups were also found by Akay et al. (2008), who detected subendothelial B2M in 63.3% cases with HP and none of the HP-negative control group (*p* < 0.001) and B2M accumulating in the majority of HP-positive patients with active chronic gastritis, whereas no correlation between the serum and tissue levels was found [52]. B2M deposition has also been found in the subepithelial layer of gastric biopsies from patients with HP-positive active chronic gastritis, with this process being associated with the significant leucocyte infiltration during HP infection and its subsequent release of B2M from their surface [53]. In our study, at gastric tissue level, we did not observe a statistically significant difference in B2M relative abundance according to the DSC score, and the B2M levels did not correlate with age in patients aged 55–70 years. However, given the small size of our tissue cohort, we cannot exclude the possibility of differential B2M deposition within inflammatory gastric tissue depending on gastric atrophy and *H. pylori* infection. In several solid cancers (i.e., prostate, breast, lung and renal cancer), in situ B2M can exert tumorigenic effects independently of MHC activating epithelial–mesenchymal transition and inducing cancer lethality and bone metastasis by interacting with the hemochromatosis-I proteins [54]. B2M can stimulate tumor cell growth/viability and suppress apoptosis by interfering with specific pathways (i.e., cAMP-dependent PAK and PI3K/AKT in prostate and renal carcinomas, respectively [55,56]). In colorectal cancer, low B2M expression correlated with aggressive behavior (immune cold environment and high-risk histologic features) and worse disease-specific survival [57].

PZP is an estrogen-dependent highly conserved glycoprotein belonging to the α-2-macroglobulin superfamily, composed by two identical disulfide-bridged subunits of around 160 kDa. It is mainly produced by the liver and expressed in extracellular compartments of several tissues, but also secreted in body fluids, such as serum (revised by Wu [58]). PZP plays fundamental roles as a broad spectrum protease activity inhibitor, immune regulator/immunosuppressant and inhibitor of misfolded protein aggregates [59,60]. During pregnancy, PZP serum levels dramatically increase, but decline rapidly postpartum, while in the serum of non-pregnant females and healthy males, PZP is a trace protein (<0.01 mg/mL) [61,62]. Circulating (plasma/serum) PZP levels can also vary (increase or decrease) in different diseases, including cancer [58]. For instance, elevated serum PZP levels were identified in uterine leiomyoma, endometrial cancer and some ovarian tumors [63], and lung adenocarcinoma with type 2 diabetes mellitus [64]. Other pathologies investigated in terms of PZP plasma level variation include metabolic diseases, such as diabetes and hypertensive disorders in pregnancy [65], and conditions involving chronic inflammation (i.e., rheumatoid arthritis, RA [66]; inflammatory bowel disease, IBD [67]).

During the last years, there has also been a particular interest in deciphering the possible role(s) of PZP as a regulator of the tumor immune microenvironment in situ, and thus, indirectly, as a candidate immunotherapy target, with its role as a potential tumor marker having been recently revisited [68]. In our study, we found a significant increase in PZP abundance in S2 tissues compared to S0. The possible relationship between PZP and GC has been exploited at transcript level by Oshima et al. (2024) in GC tissues and adjacent normal gastric mucosa of 253 patients with Stage II/III GC who underwent curative resection [69]. The authors proposed the clinical utility of PZP as an independent predictor of poor OS (hazard ratio = 1.984, 95% confidence interval = 1.307–3.012, *p* = 0.0013), with its expression in GC tissues resulting in an association with differentiated histological types, venous invasion, and pathological stage. Previous studies in patients with either hepatocellular carcinoma or lung cancer reported low PZP expression in tissues where the protein was proposed as a potential prognostic indicator and tumor suppressor [70,71].

To our knowledge, at present the possible relationship between gastric atrophy or dysplasia and PZP is still to be investigated. When autoimmune, atrophic gastritis is accompanied by an increase in lymphocytic infiltrates (predominantly T cells and plasma cells) in the gastric submucosa and lamina propria and production of high levels of different pro-inflammatory cytokines, such as IFN-γ and TNF-α by CD4+ Th1 cells [72,73]. Chronic inflammation in gastric mucosa is also a typical hallmark of HP-induced gastritis [74], which notably presents increasing PGII serum levels [75]. The inhibitory role played by PZP during pregnancy is known to selectively modulate T-cell activation, particularly by suppressing Th1-type immune responses [59,71], while the lower serum PZP levels observed in S2 patients may stem from possible gastric inflammation and mucosal atrophy, potentially triggered by the autoimmunity or HP infection alongside environmental changes in the gastric milieu. The higher PZP abundance we observed in S2 gastric tissues may be related to a sequestration mechanism counteracting local immune responses. The serum PZP levels were not significantly associated with any of the serological parameters that were individually tested, but they showed a significant association with sex, being higher in female than in male. This finding is consistent with the DSC screening test, which associated the S2 score and the higher risk of developing gastric pathology with the male sex, and with epidemiological evidence reporting a higher GC incidence in males [76].

To gain better insight into the potential contribution of PZP to altered local and systemic immune responses, future studies should investigate PZP expression in gastric tissues and correlate its levels with those of pro-inflammatory cytokines. Within this context at tissue level, we identified preliminary differences in the inflammatory signature between S0 and S2 patients, potentially indicating that our serological classification is associated with distinct mucosal immune profiles. An additional role played by PZP may also be in tissue remodeling as a protease inhibitor; this may also be the case for some S2 patients who are at risk for precancerous lesions’ development into intestinal-type gastric cancer.

Among the other differentially abundant proteins that increased in S2 serum, the extracellular matrix remodeler EFEMP1 has recently emerged as having a pro-tumorigenic role in gastric carcinogenesis [77], and the thiol proteinase inhibitor CST3 may function as either a tumor suppressor or a tumor promoter [78], while CFD acts as both a signaling protein and, in the specific context of GC, as an effective auxiliary biomarker for early-stage screening [79]. Conversely, among the proteins decreased in S2 serum is SHBG, a sex steroid hormone-binding protein that was traditionally associated with chronic atrophic gastritis [80] and GC in men [81].

Given the biological relevance of B2M and PZP, together with their statistical association with the DSC score, we focused our analysis on these candidate biomarkers as complementary components of the DSC model. Replacing age and sex with these two proteins resulted in a modest improvement in classification performance. Notably, age remained significantly associated with the outcome, indicating a relevant contribution that was consistent with the correlation between the B2M levels and aging observed in this study. However, the persistence of this association may also suggest that B2M and PZP contribute to the model beyond the independence of the age-related effect alone. Furthermore, the B2M/PZP-related model outperformed in the AUC and accuracy compared with the standard version, supporting their potential added value in risk stratification.

Our study has several limitations. First, the relatively small sample size of the selected patient cohorts may only partially capture the vast heterogeneity of the serum proteome. Nevertheless, our findings provide a robust proof-of-concept for the role of PZP and B2M in gastric disease risk stratification. Future large-scale, multi-center studies are warranted to validate these biomarkers and further assess their clinical utility. Specifically, further analyses stratified according to key clinical parameters—such as gastric atrophy, intestinal metaplasia, and dysplasia—may help to better define diagnostic performance across different stages of gastric precancerous progression.

Another limitation is the inherent complexity of the serum matrix; protein profiles vary considerably based on factors such as age, sex, hormonal status, and metabolism. Since PZP and B2M levels are significantly influenced by these demographic and metabolic factors, such variability highlights the need for tailored reference intervals to ensure diagnostic accuracy and minimize false results. Furthermore, fluctuations in circulating PZP and B2M reflect local or systemic responses that are not specific to gastric pathology. While our findings demonstrate a strong correlation between these markers and an increased risk of gastric disease (DSC score 2), further research is needed to elucidate their exact mechanistic relevance and determine whether this altered protein signature directly contributes to GC development.

Future objectives could include an in situ investigation of B2M and PZP levels in S2 patients with extreme protein abundance (highest vs. lowest) to gain deeper insight into their roles as markers for precancerous conditions. Additionally, in line with our identification of novel early GC risk markers, further studies should investigate whether other proteins identified in this study—such as the matrix-related EFEMP1 or metabolic markers SHBG and CFD—may further improve the diagnostic performance of the test. Finally, exploring the PD-1/PD-L1 axis or other immune regulatory pathways, including the emerging checkpoint molecule Siglec-15 [82] may help to contextualize the observed proteomic alterations within the broader context of tumor-associated immune modulation.

## 4. Materials and Methods

### 4.1. Patient Selection

The study enrolled consecutive patients undergoing esophagogastroduodenoscopy and gastric biopsy at the Unit of Oncological Gastroenterology of Centro di Riferimento Oncologico di Aviano (CRO) between April 2014 and December 2016. Patients whose sera were analyzed gave written informed consent. The study protocol no. CRO-2024-04 was approved by the Ethical Committee of Friuli Venezia Giulia (CEUR-2024-Os-128/16 July 2024) and registered at ClinicalTrials.gov (ID NCT07374731). The study was conducted in compliance with the Declaration of Helsinki principles and Good Clinical Practice. Written informed consent was obtained from all participants. The inclusion criteria were: age over 18 years; ability to understand, accept, and sign informed consent for the study, data processing and the collection of a serum aliquot for research purposes; and fasted subjects with suspected pre-neoplastic/neoplastic gastric lesion or with a request for pepsinogen testing, for whom endoscopic examination data are available. The exclusion criteria were: pregnant women; patients with co-existing or prior diagnosis (within the last 5 years) of another malignant neoplasm; and patients who were unable to understand, accept, and sign consent for data processing. Based on their DSC score, individuals were grouped into “score 0” (S0) or “score 2” (S2), indicating a “low risk” or “high risk” for developing severe gastric diseases, including GC [21].

### 4.2. Study Design

The patients were divided into four groups: (i) a discovery group of score 0 (*n* = 10); (ii) a discovery group of score 2 (*n* = 10); (iii) a validation group of score 0 (*n* = 51); and (iv) a validation group of score 2 (*n* = 29). Our discovery and validation cohorts consisted of the two discovery and two validation groups, respectively. Label-free LC-MS/MS was used to analyze and compare the two groups (S0 and S2) in the discovery cohort in Part I of the study, and in the validation cohort in Part II. The relative abundance of some selected proteins was found to differ between the two groups in both cohorts at serum level. To further investigate these findings at tissue level, we analyzed an additional independent cohort of patients with scores 0 (*n* = 7) and 2 (*n* = 7). Atrophic gastritis was respectively excluded (score 0) or confirmed (score 2) in these subjects via esophagogastroduodenoscopy and histology.

### 4.3. Label-Free Serum Proteomic Profiling by LC-MS/MS

The proteins were reduced, alkylated, and digested, and the peptides were cleaned up with the EasyPep Mini MS Sample Prep Kit (Thermo Fisher Scientific, Waltham, MA USA) according to the manufacturer’s protocol. The concentration of peptides resuspended in 0.1% formic acid was measured with the Pierce BCA Protein Assay Kit (Thermo Fisher Scientific). The peptide mixtures were analyzed using a Q-Exactive Plus Hybrid Orbitrap mass spectrometer (Thermo Fisher Scientific) equipped with a UHPLC Vanquish (Thermo Fisher Scientific), as previously reported [83]. Briefly, each tryptic peptide sample (15 μg) was fractionated in a XBridge Peptide BEH C18 column (3.5 µm 2.1 × 150, Waters, Sesto San Giovanni, Milan, Italy) at a flow rate of 200 µL/min, using 0.1% formic acid/acetonitrile gradient over a period of 80 min, and sprayed onto the mass spectrometer using a heated electrospray source probe in positive mode (Thermo Fisher Scientific). The mass spectrometer was run in data-dependent mode with positive polarity at an electrospray voltage of 3.52 kV and capillary temperature of 325 °C. Full scan MS spectra (*m*/*z* 375–1500) were acquired followed by MS/MS scans on the top 10 intense ions, applying a dynamic exclusion window of 30 s. Individual samples of the exploratory cohort were run in single runs, while those from the validation cohort were in duplicate. Commercially available peptides from tryptic digested HeLa proteins (Thermo Fisher Scientific) were used as quality control standards. The label-free quantification (LFQ) and database search were done with Proteome Discoverer (PD) software (version 2.5.0.400), using the Sequest search engine against the human database (UniProt release 2025_04). Only proteins identified with a false discovery rate (FDR) that was high (1%), a Sequest Score ≥ 1, and a *t*-test *p*-value < 0.05 were considered. The abundance ratio (or fold change, FC) of statistically significant proteins was calculated as the ratio of the average LFQ intensities between the two matched groups, with the differential proteins increasing or decreasing in abundance depending on the FC values ≥ 1.5 (FC Log2 ≥ 0.6) or ≤1.5 (FC Log2 ≤ −0.6), respectively.

### 4.4. Validation of Candidate Markers by Immunoblotting

Plasma pools (S0 and S2) were treated with a 2D Clean-Up Kit (Cytiva, Uppsala, Sweden) and centrifuged. The resulting pellets were resuspended in lysis buffer (Thermo Fisher Scientific). The protein concentration was determined with the Pierce BCA Protein Assay Kit (Thermo Fisher Scientific). Samples were stored at −80 °C until analysis. Protein (30 and 50 µg per pool for pregnancy zone protein PZP and beta-2-microglobulin B2M, respectively) was fractionated on 4–20% gradient Criterion TGX Stain-Free gels (Bio-Rad Laboratories, Hercules, CA, USA). Gel images were acquired with the Chemidoc system (Bio-Rad) to document equal protein loading among samples. Then, separated proteins were electrotransferred onto nitrocellulose membranes and processed with the PURITY™ WB horseradish peroxidase (HRP) Detection System (Vilber, Eberhardzell, Germany), according to the manufacturer’s protocol. The protein bands were visualized by chemiluminescence using SuperSignal West Femto Substrate Maximum Sensitivity Substrate (Thermo Fisher Scientific) and imaged with ChemiDoc (Bio-Rad). The following primary antibodies were tested: anti-PZP (1:1000; #PA5-110249, Thermo Fisher Scientific) and anti-B2M (1:500; #ab14714, AbCam, Waltham, MA, USA).

### 4.5. Tissue Proteomic Profiling by LC-MS/MS

Protein extracts from gastric tissue (S0 and S2 samples), solubilized in 7 M urea, 2 M thiourea, and 4% (*w*/*v*) CHAPS, were reduced, alkylated, and digested. The resulting peptides were cleaned up and separated in duplicate, as described above. After raw data interrogation with PD software (version 2.5.0.400), as described above, we calculated the abundance ratio of B2M and PZPs in S2 vs. S0 groups (significant *t*-test *p*-value < 0.05; FC Log2 ≥ 0.6 or FC Log2 ≤ −0.6).

### 4.6. Statistics and DSC Score Classification Accuracy

Continuous variables were summarized as mean ± standard deviation or mean and interquartile range, according to data distribution, while categorical variables were reported as counts and percentages. Demographic and serological variables in the discovery and validation cohorts were compared using Fisher’s exact test and Mann–Whitney as appropriate. Correlations between variables were evaluated using the Mann–Whitney U test and Spearman’s rank correlation coefficient. The statistical significance was set at *p* < 0.05. To evaluate the classification performance of the DSC score, subjects were categorized according to the predefined DSC class score (0 and 2). Classification accuracy was assessed using receiver operating characteristic (ROC) curve analysis, and the area under the curve (AUC) was calculated as a measure of model discrimination. Accuracy, sensitivity, specificity, and positive/negative predicted value were additionally estimated. Multivariate logistic regression models were implemented to assess the contribution of demographic variables and candidate proteomic biomarkers, replacing age and sex with B2M and PZP, to the DSC-based stratification. Model performance was compared between the standard DSC model and biomarker-added versions using AUC and overall classification accuracy. All statistical analyses were performed using R (version 4.2.2) and MedCalc (version 22.032) software.

## 5. Conclusions

In two independent cohorts classified as S0 and S2 by our DSC screening approach, proteomic analysis identified increased B2M and decreased PZP serum levels in patients who were at high risk of gastric pathology. These findings reinforce the biological relevance of the DSC classification, particularly in higher-risk demographic groups such as older males. Integration of these proteomic signatures into the DSC framework may improve risk stratification, potentially enabling more personalized surveillance strategies and earlier intervention in high-risk individuals. However, these findings remain preliminary and require validation in larger cohorts to better define their clinical biological relevance.

## Figures and Tables

**Table 1:** Demographic and serological data in the discovery and validation cohorts.

	Discovery Cohort	Validation Cohort	
	*n* = 20	*n* = 80	*p*-Value
Class age < 50 y (%)	5 (25)	41 (51)	0.045
50–70 y (%)	8 (40)	14 (18)	0.039
>70 y (%)	7 (35)	25 (31)	0.792
Gender, male (%)	7 (35)	26 (33)	1
*Helicobacter pylori* IgG > 30 EIU (%)	3 (16)	5 (6)	0.178
PGI ng/mL, median (IQR)	93 (53–126)	90 (75–103)	0.73
PGII ng/mL, median (IQR)	11 (10–16)	11 (8–15)	0.476
PGR, median (IQR)	8 (6–12)	10 (8–13)	0.134
G17 pmol/L, median (IQR)	5 (3–10)	4 (3–10)	0.683

EIU, Enzyme Immune Units; PGI, pepsinogen I; PGII, pepsinogen II; PGR, pepsinogen I to pepsinogen II ratio; and G17, gastrin-17.

**Table 2:** Differences in the abundance of proteins in the serum of individuals by DSC score (S2 vs. S0) in the discovery cohort (*p* < 0.05).

Acc.	Description	Gene Symbol	Score Sequest HT:	FC (Log2): (S2)/(S0)	FC *p*-Value: (S2)/(S0)	FC Adj. *p*-Value: (S2)/(S0)	Ab. S0	Ab. S2	Ab. CV [%]: S0	Ab. CV [%]: S2
**More abundant in S2 (*n* = 7)**										
P06331	Immunoglobulin heavy variable 4-34	IGHV4-34	361.90	1.4	0.0398	0.4079	55.9	144.1	71.50	62.02
Q12805	EGF-containing fibulin-like extracellular matrix protein 1 °	EFEMP1	25.97	1.2	0.0023	0.1314	59.5	140.5	56.23	56.98
P61769	Beta-2-microglobulin °	B2M	115.81	1.2	5.05 × 10^−5^	0.0068	61.4	138.6	37.54	17.51
P00746	Complement factor D °	CFD	70.48	1.1	4.58 × 10^−5^	0.0068	64.7	135.3	37.40	25.69
P01034	Cystatin-C °	CST3	8.60	1.0	0.0351	0.4079	68.2	131.8	58.68	38.43
A0A075B6S5	Immunoglobulin kappa variable 1-27	IGKV1-27	117.64	0.9	0.0100	0.2692	70.7	129.3	51.34	43.63
P18428	Lipopolysaccharide-binding protein	LBP	157.77	0.8	0.0194	0.3275	72.9	127.1	33.98	39.33
**More abundant in S0 (*n* = 7)**										
Q9UGM5	Fetuin-B	FETUB	148.12	−0.6	0.0024	0.1314	119.3	80.7	29.85	24.86
P03951	Coagulation factor XI	F11	12.58	−0.7	0.0023	0.1314	124.3	75.7	22.77	33.13
P27169	Serum paraoxonase/arylesterase 1	PON1	1020.78	−0.7	0.0048	0.2000	125.1	74.9	25.30	39.52
P35858	Insulin-like growth factor-binding protein complex acid labile subunit	IGFALS	564.07	−0.8	3.87 × 10^−5^	0.0067	127.7	72.3	20.08	27.34
A5A3E0	POTE ankyrin domain family member F	POTEF	17.73	−0.9	0.0361	0.4078	128.8	71.2	32.03	40.91
P04278	Sex hormone-binding globulin °	SHBG	309.79	−1.1	0.0092	0.2619	137.7	62.3	69.30	58.49
P20742	Pregnancy zone protein °	PZP	2448.02	−2.2	0.0129	0.2739	164.3	35.7	171.08	103.30

° difference in protein abundance confirmed in the validation cohort (Table 3); Acc., UniProt Knowledgebase accession number; Ab., abundance; and FC, fold change (LFQ intensity ratio between S2 and S0).

**Table 3:** Differences in the abundance of proteins in the serum of individuals by DSC score (S2 vs. S0) in the validation cohort (*p* < 0.05).

Acc.	Description	Gene Symbol	Score Sequest HT:	FC (Log2): (S2)/(S0)	FC *p*-Value: (S2)/(S0)	FC Adj. *p*-Value: (S2)/(S0)	Ab. S0	Ab. S2	Ab. CV [%]: S0	Ab. CV [%]: S2
**More abundant in S2 (*n* = 13)**										
P02675	Fibrinogen beta chain	FGB	23.61	1.35	0.0006 1	0.008814037	56.4	143.6	184.45	71.58
P08519	Apolipoprotein(a)	LPA	2876.20	1.32	0.0400	0.182228738	57.3	142.7	125.62	102.8
P07998	Ribonuclease pancreatic	RNASE1	30.74	0.95	0.0004	0.005736136	68.2	131.8	77.57	62.48
P02679	Fibrinogen gamma chain	FGG	47.87	0.86	0.0038	0.038226774	70.9	129.1	128.83	48.20
P00746	Complement factor D	CFD	421.54	0.84	3.1088 × 10^−9^	9.80828 × 10^−7^	71.8	128.2	36.02	34.98
Q9NQ79	Cartilage acidic protein 1	CRTAC1	114.32	0.71	3.27164 × 10^−5^	0.001056618	76.0	124.0	55.67	45.09
P61769	Beta-2-microglobulin	B2M	493.31	0.70	1.19552 × 10^−8^	2.075 × 10^−6^	76.1	123.9	28.39	34.61
Q12805	EGF-containing fibulin-like extracellular matrix protein 1	EFEMP1	250.78	0.68	1.17896 × 10^−6^	0.000106275	76.8	123.2	50.14	32.62
P0DJI9	Serum amyloid A-2 protein	SAA2	171.40	0.64	0.0253	0.137709754	78.2	121.8	387.24	117.58
Q92954	Proteoglycan 4	PRG4	231.91	0.63	0.0006	0.009371515	78.4	121.6	57.05	36.88
O75460	Serine/threonine-protein kinase/endoribonuclease IRE1	ERN1	376.58	0.59	0.0009	0.012633807	79.7	120.3	47.32	37.62
Q02985	Complement factor H-related protein 3	CFHR3	746.82	0.58	0.0392	0.180446882	80.2	119.8	63.18	58.96
P01034	Cystatin-C	CST3	288.43	0.58	0.0012	0.014245287	80.3	119.7	37.17	42.23
**More abundant in S0 (*n* = 8)**										
P14151	L-selectin	SELL	21.06	−0.55	0.0018	0.020011824	118.7	81.3	39.87	38.02
A0A0G2JS06	Immunoglobulin lambda variable 5-39	IGLV5-39	44.61	−0.62	0.0039	0.039217887	121.2	78.8	59.70	59.55
P01705	Immunoglobulin lambda variable 2-23	IGLV2-23	86.94	−0.64	0.0431	0.191508477	121.8	78.2	52.82	67.65
P04211	Immunoglobulin lambda variable 7-43	IGLV7-43	692.58	−0.81	0.0004	0.006648553	127.2	72.8	46.28	48.86
P01704	Immunoglobulin lambda variable 2-14	IGLV2-14	95.38	−0.84	0.0027	0.029178574	128.3	71.7	61.53	57.76
P04278	Sex hormone-binding globulin	SHBG	2369.20	−0.92	0.0015	0.018350946	130.8	69.2	105.91	60.86
P20742	Pregnancy zone protein	PZP	19422	−0.95	0.0010	0.012633807	131.6	68.4	234.45	107.22
P09172	Dopamine beta-hydroxylase	DBH	188.40	−1.28	0.0033	0.034356657	141.8	58.2	69.46	95.01

Acc., UniProt Knowledgebase accession number; Ab., abundance; and FC, fold change (LFQ intensity ratio between S2 and S0).

**Table 4:** Correlation between B2M and PZP levels and serological parameters.

	B2M		PZP	
Serological Parameter	r	*p*-Value	r	*p*-Value
Sex	26.000	0.122	5.000	<0.001
Age	0.659	0.002	−0.205	0.387
PGI	0.159	0.502	−0.259	0.271
PGII	0.104	0.671	−0.079	0.748
PGI/PGII	0.116	0.627	−0.277	0.238
G17	0.111	0.643	0.103	0.666
HP IgG	−0.026	0.913	−0.322	0.166

G17, gastrin-17; HP, *Helicobacter pylori*; PG I, pepsinogen I; and PG II, pepsinogen II.

## Data Availability

The original contributions presented in this study are included in the article/Supplementary Material. Further inquiries can be directed to the corresponding authors.

## Supplementary Materials

The following supporting information can be downloaded at: https://www.mdpi.com/article/10.3390/ijms27104464/s1.
