# Supplementary figures and images for: Serum Protein Profiling of Patients at Risk to Develop Gastric Disease Based on a DSC Test
Source: Int J Mol Sci. 2026 May 16;27(10):4464. doi: 10.3390/ijms27104464 (PMC13206807; doi:10.3390/ijms27104464)

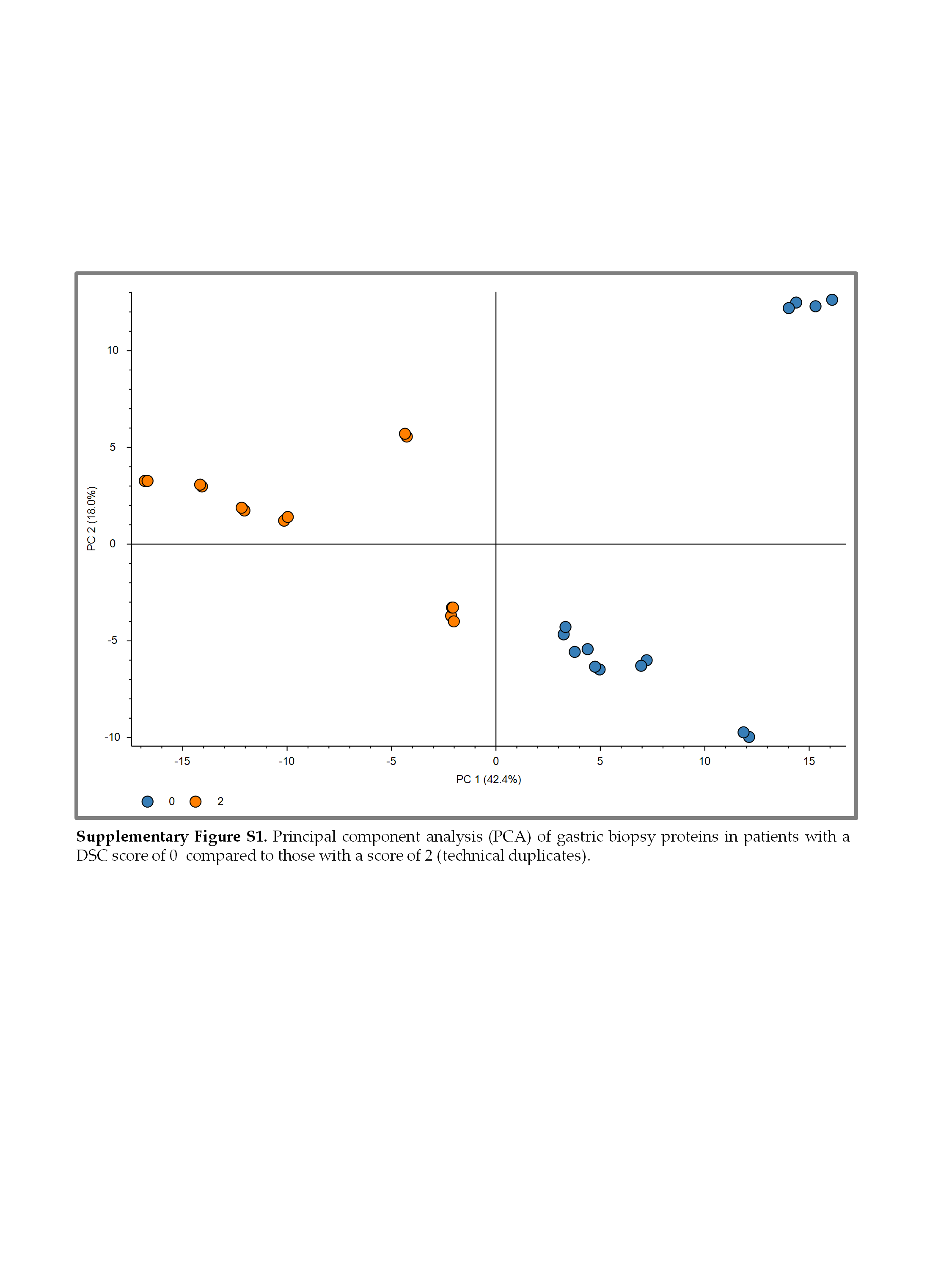

Supplement: Supplementary file 1 [file ijms-27-04464-s001.zip › Figure S1.tif]

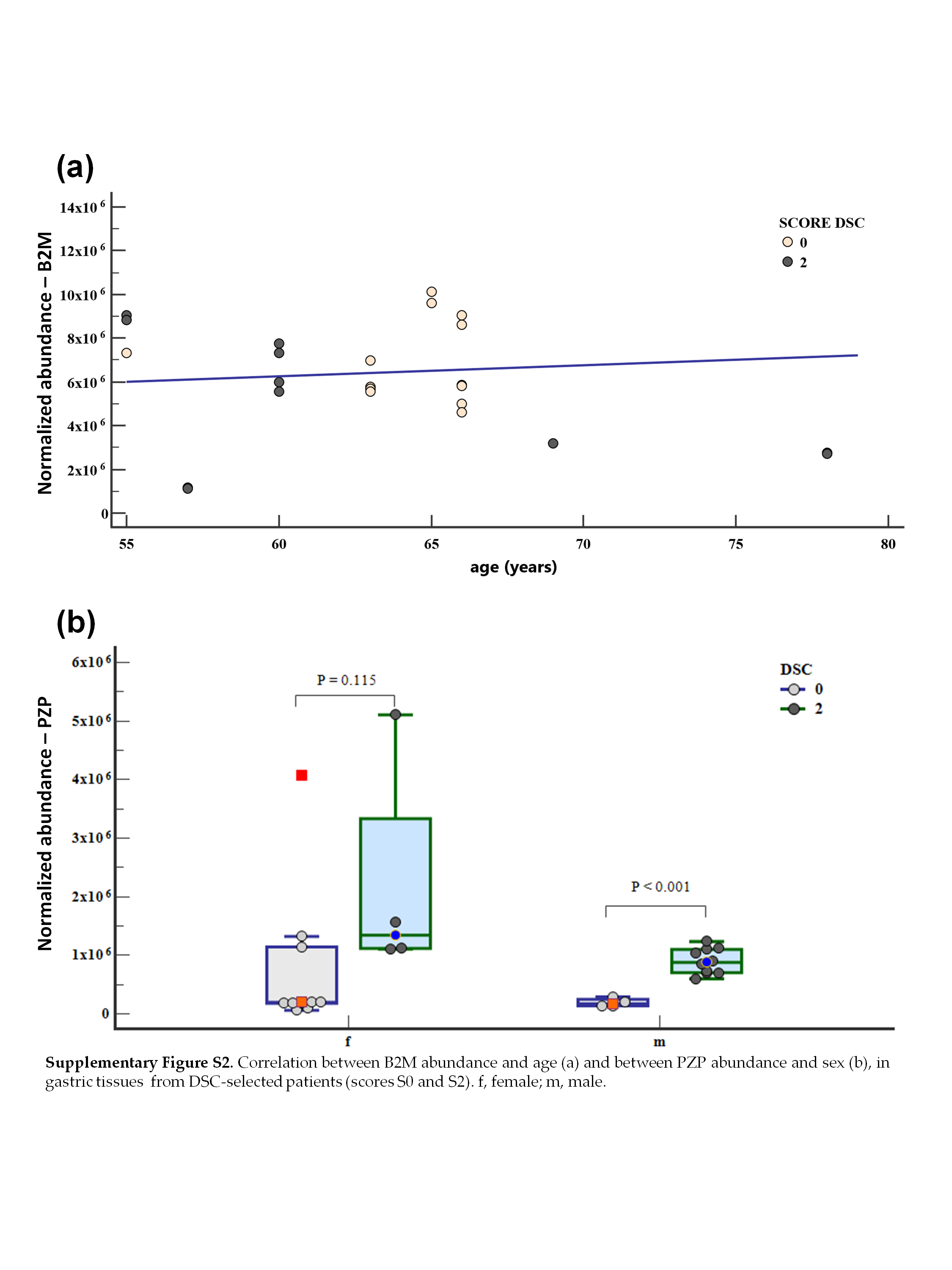

Supplement: Supplementary file 1 [file ijms-27-04464-s001.zip › Figure S2.tif]

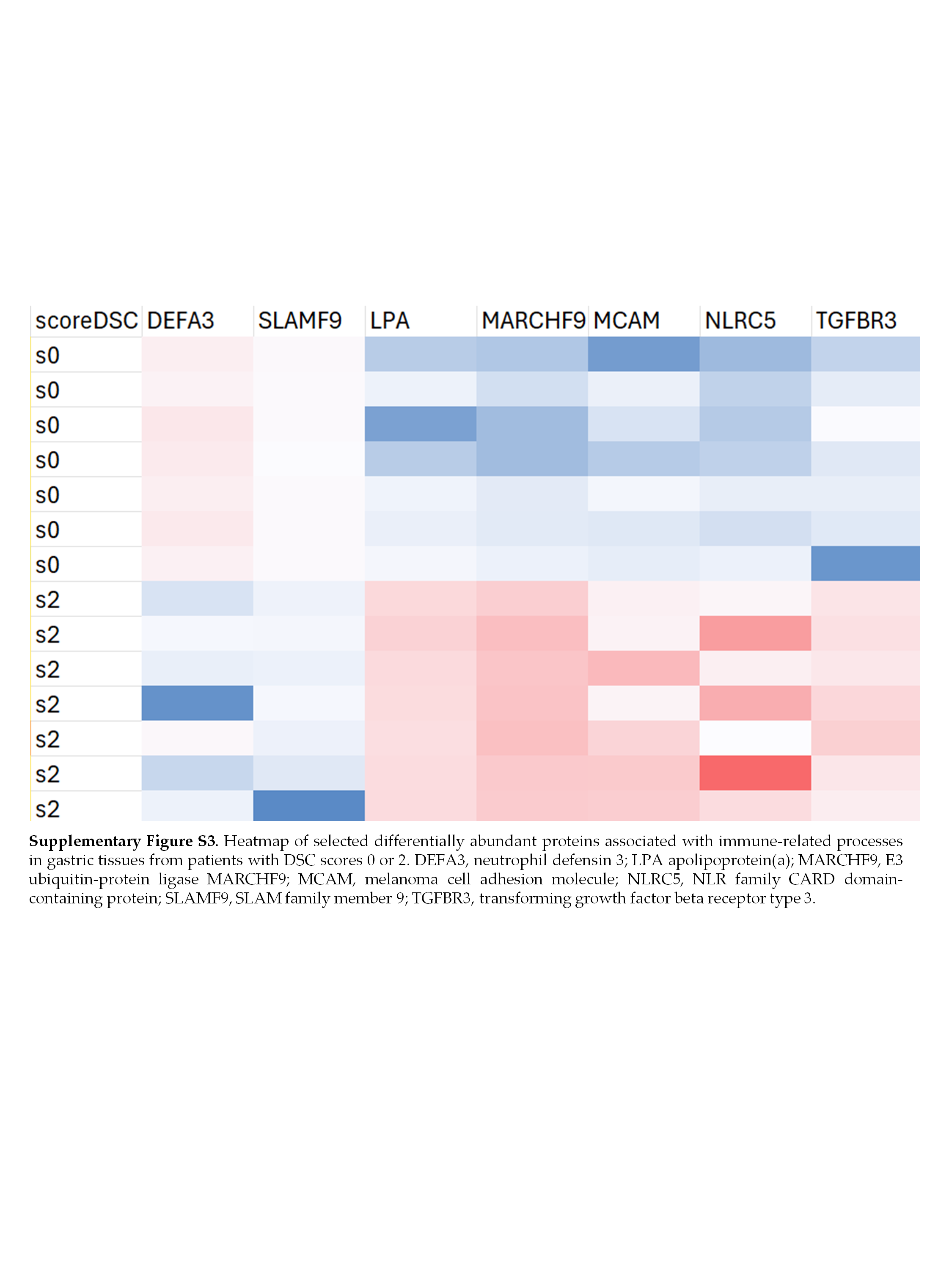

Supplement: Supplementary file 1 [file ijms-27-04464-s001.zip › Figure S3.tif]

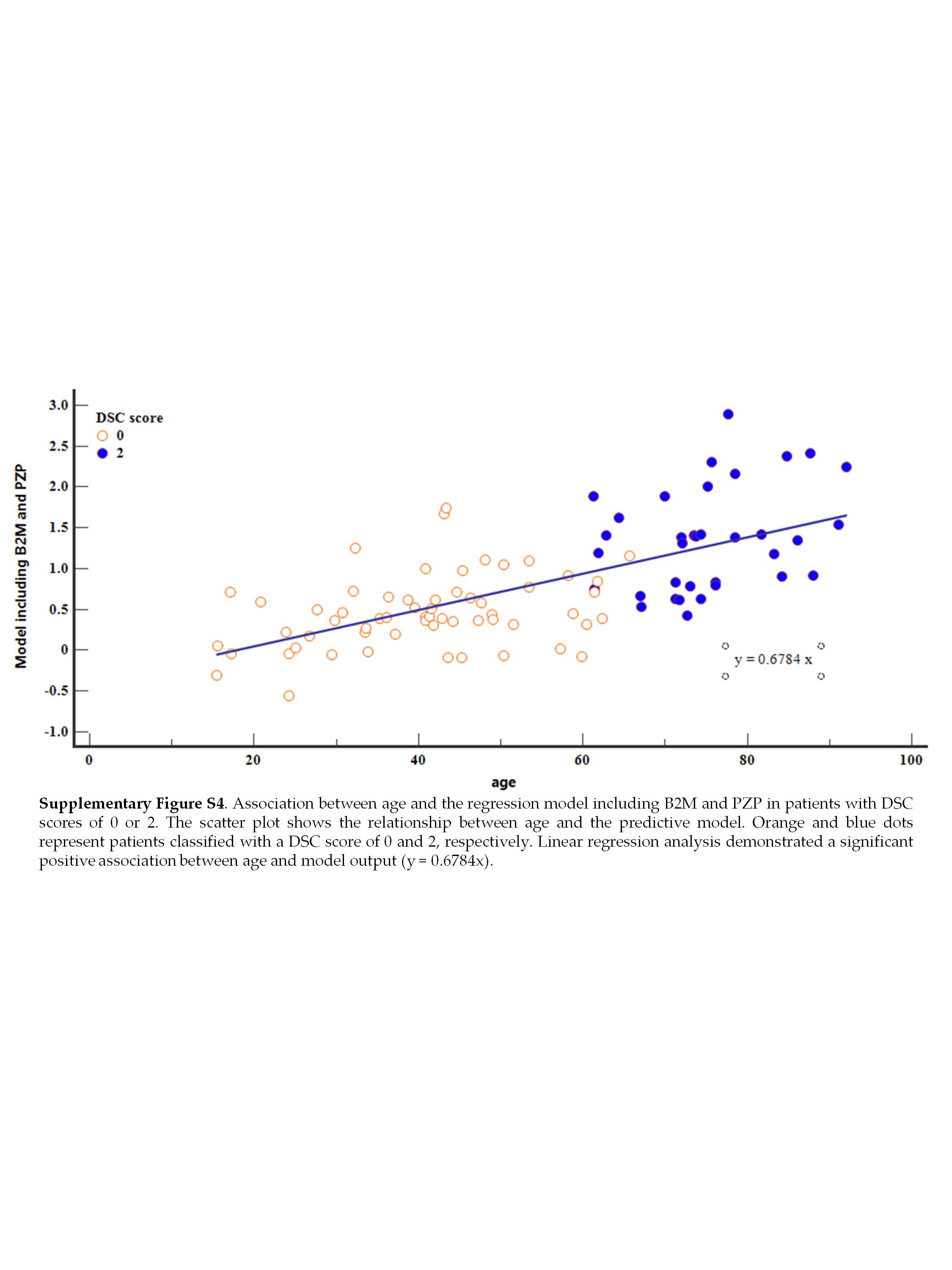

Supplement: Supplementary file 1 [file ijms-27-04464-s001.zip › Figure S4.tif]

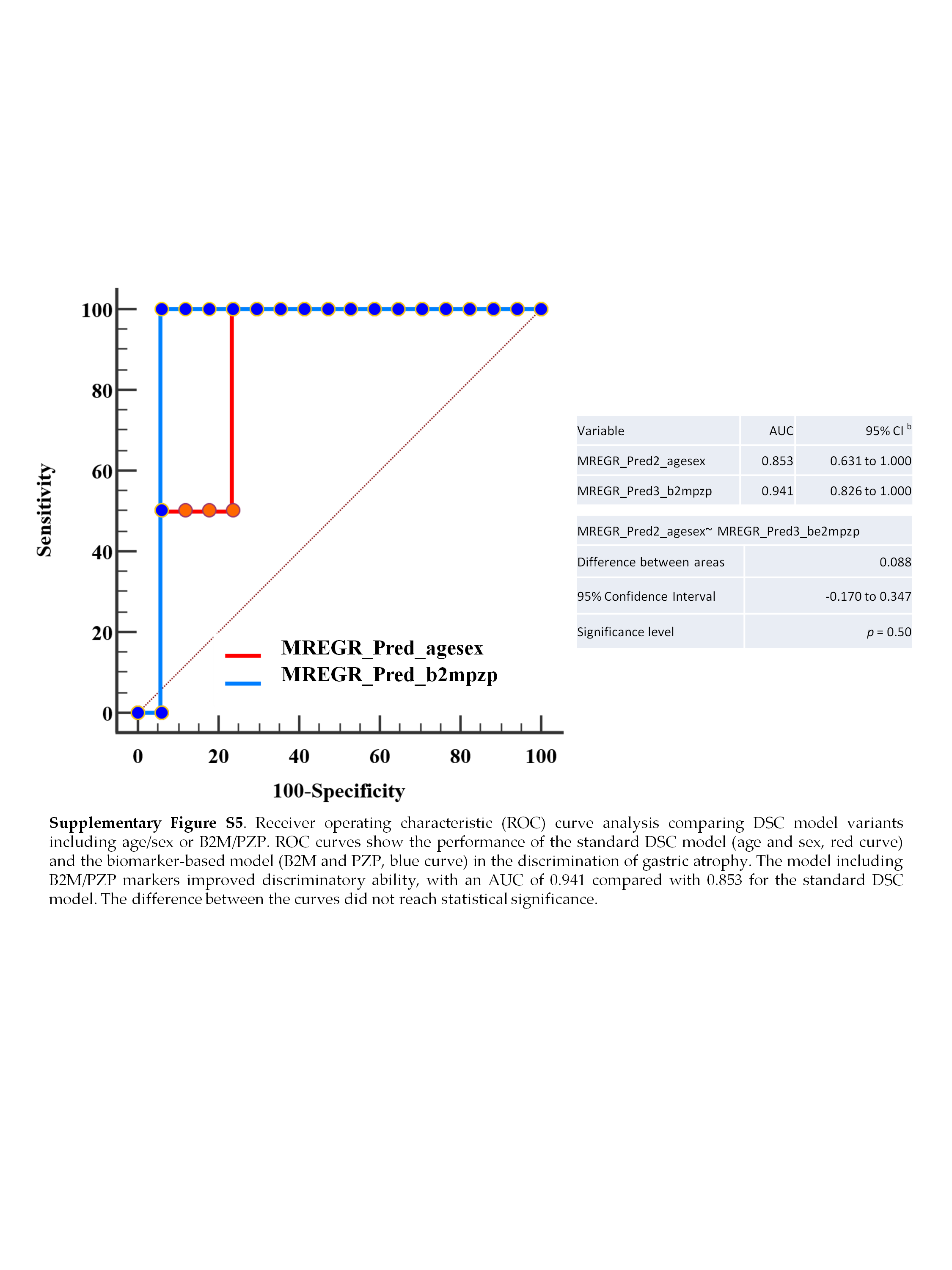

Supplement: Supplementary file 1 [file ijms-27-04464-s001.zip › Figure S5.tif]
